# Boulder Dash: Endoscopic Management of Bouveret Syndrome and Gallstone Ileus

**DOI:** 10.14309/crj.0000000000001530

**Published:** 2024-10-18

**Authors:** William Hirsch, Bryant Megna, Nabeel Azeem

**Affiliations:** 1 Department of Medicine, University of Minnesota, Minneapolis, MN; 2 Department of Medicine, University of Minnesota, Division of Gastroenterology, Hepatology, and Nutrition, Minneapolis, MN

**Keywords:** bouveret syndrome, gallstone ileus, gallstone disease

## CASE REPORT

**Figure 1.**
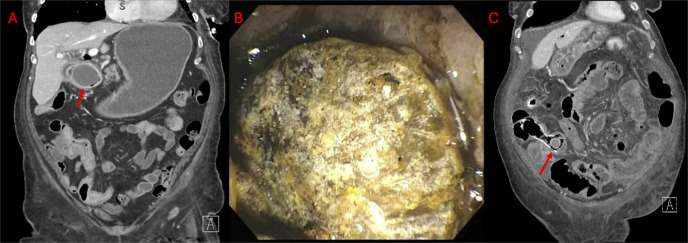
(A) Coronal CT abdomen and pelvis demonstrating gallstone within the duodenal bulb and resulting gastric distention. (B) Gallstone located at the duodenal bulb visualized on EGD. (C) Coronal CT abdomen and pelvis demonstrating gallstone fragment within the small bowel leading to small bowel obstruction.

A 68-year-old woman with metastatic colon adenocarcinoma presented with 2 weeks of nausea and emesis. She was nontoxic appearing with soft, nontender, and nondistended abdomen. A computed tomography (CT) scan demonstrated a cholecystoduodenal fistula with migration of a 3.0 × 5.1 cm gallstone into the duodenal bulb with associated gastric distention, consistent with Bouveret syndrome (Figure 1). The cholecystoduodenal fistula was suspected to have developed spontaneously, possibly exacerbated by the prior use of bevacizumab.

An esophagogastroduodenoscopy showed an obstructing stone in the duodenal bulb (Figure 1). Endoscopic electrohydraulic lithotripsy was used to fragment the stone; large fragments >1.5 cm were subsequently removed with a retrieval net while smaller fragments were irrigated into the downstream duodenum. Recurrent symptoms prompted a repeat CT showing distal migration of stone fragments causing ileal obstruction. Colonoscopy identified a malignant ileocecal valve stricture. A 25 × 90 mm uncovered metal stent was placed across the stricture with subsequent flow of small gallstone fragments. Repeat CT showed gallstone fragments in the proximal end of the stent with upstream small bowel dilation (Figure 1). Repeat colonoscopy visualized stone fragments in the left colon, which were removed. The previously placed stent had expanded allowing traversal, and the obstructing stone fragment was removed.

This case illustrates the successful endoscopic management of a unique presentation of Bouveret syndrome with subsequent gallstone ileus.

## DISCLOSURES

Author contributions: W. Hirsch: draft writing, draft review, organization, inception; B. Megna: inception, draft review; N. Azeem: draft review, project oversight, final approval, and is the article guarantor.

Financial disclosure: N. Azeem: consultant for Boston Scientific.

Previous presentation: This project was presented as a poster at the ACG 2023 Annual Scientific Meeting; October 24, 2023; Vancouver, BC, Canada.

Informed consent was obtained for this case report.

